# Clinical effectiveness of Finger gliding Exercise for patients with trigger fingers receiving steroid injection: a Randomized Clinical Trial

**DOI:** 10.1038/s41598-025-89436-9

**Published:** 2025-02-11

**Authors:** Yue Kwan Choi, Regina Wing-Shan Sit, Bo Wang, Christina Cheuk, Man Kei Lee, Kwan Wa Maria Leung

**Affiliations:** 1 Department of Family Medicine, New Territories East Cluster, Hospital Authority, New Territories, Hong Kong; 2 Hong Kong Jockey Club School of Public Health and Primary Care, The Chinese University of Hong Kong, New Territories, Hong Kong

**Keywords:** trigger finger, finger gliding exercises, steroid injection, clinical effectiveness, Health care, Pain management, Outcomes research, Musculoskeletal abnormalities, Tendons

## Abstract

Finger gliding exercises are believed to enhance flexor tendons excursion. This study assesses the effectiveness of finger gliding exercises in patients after receiving steroid injections for trigger fingers. Patients with trigger fingers who received corticosteroid injection were randomly assigned (1:1) to control and intervention group. The intervention group was required to do finger exercises and submit online exercise log regularly. The clinical outcomes of trigger fingers at 24 weeks and compliance with finger gliding exercises were assessed via online surveys. A total of 38 participants were allocated to each group. Baseline characteristics were similar, except for a longer duration of symptoms in the intervention group (5.2 ± 2.9 vs. 3.6 ± 2.6 months, *P* = 0.002). At 24 weeks, 34 (89.5%) control and 33 (86.8%) intervention group participants responded to online survey. No statistical significant differences were observed in Numerical Pain Rating Score, Quinelle grading, finger improvement rate, recurrence of triggering, need for repeated injection and occurrence of new trigger finger sites. The exercise log response rate and compliance rate were 85.6% and 68.6%. In conclusion, our study did not establish the clinical effectiveness of finger gliding exercise for trigger finger patients following steroid injections compared to usual care.

**Supplementary Information:**

The online version contains supplementary material available at 10.1038/s41598-025-89436-9.

## Introduction

Trigger finger (stenosing tenosynovitis) is a common musculoskeletal condition in primary care. It is thought to be due to inflammation and subsequent stenosis of the A1 pulley which leads to pain, clicking and even locking of the affected finger^1^. It has a life time risk of 2.6% and is the fourth most common reason for referral to hand surgeon^2,3^. As normal hand function is essential for daily activity and function, trigger finger can be a frustrating and disabling condition that negatively impact the quality of life^4^. Corticosteroid injection has been the first-line treatment of trigger fingers and its successful rate ranges from 67 to 90% after the first injection^5–7^. However, studies have shown that the recurrent rate of trigger fingers ranges from 11 to 56%, with most recurrence occurs within the first one year^8–14^. Although risk factors for recurrent trigger fingers such as diabetes, carpal tunnel syndrome (CTS) and multiple trigger fingers have been identified, study on strategies to prevent recurrent has been lacking^15^.

Finger gliding exercises aims to increase excursion of flexor tendons and maintain full range of movement of fingers^16^. The digits other than thumb have 2 flexor tendons under the tendon sheath, the flexor digitorum superficialis (FDS) and flexor digitorum profundus (FDP). For thumb, only the flexor pollicis longus (FPL) run under the tendon sheath^17^. In trigger finger patients, the flexor tendons are caught at the proximal portion of A1 pulley when the digit is fully flexed. Finger flexor tendon gliding exercises can allow maximum excursion of the individual flexor tendon in respect to each other and to bone and to flexor sheath^18,19^. The exercises also force each of the digital joints to glide through its full potential range. It can ensure smooth movement of finger flexor tendons under A1 pulley with potential of preventing formation of adhesions and inflammatory or degenerative process of flexor tendons.

The clinical effect of finger gliding exercises has been mostly studied in patients with CTS. However, for most of these studies, the finger tendon gliding exercises are performed together with nerve gliding exercises and they typically demonstrate effectiveness when combined with other conservative treatments. A systemic review of 4 randomized controlled trials suggests that finger tendon and nerve gliding exercises, when combined with conventional treatments, may have a favourable effect in patients with CTS^20^. As trigger finger is due to reduced space of the A1 pulley resulting in friction between the retinacular sheath and flexor tendon, it is believed that maintaining smooth sliding motion between the pulley and flexor tendon is essential for maintaining normal function of hand^1,21^. Despite fingers gliding exercises being suggested for patients with trigger fingers, their effectiveness for treating or preventing recurrence of trigger fingers have not been studied^22–24^.

The aim of this study was to evaluate the clinical effectiveness of finger gliding exercises in preventing recurrent of trigger finger symptoms patients who have received steroid injection for their trigger finger. The pain score, Quinelle grading, self-reported recurrence of trigger finger symptoms, and occurrences of new trigger finger will be assessed.

## Results

**Fig. 1:**
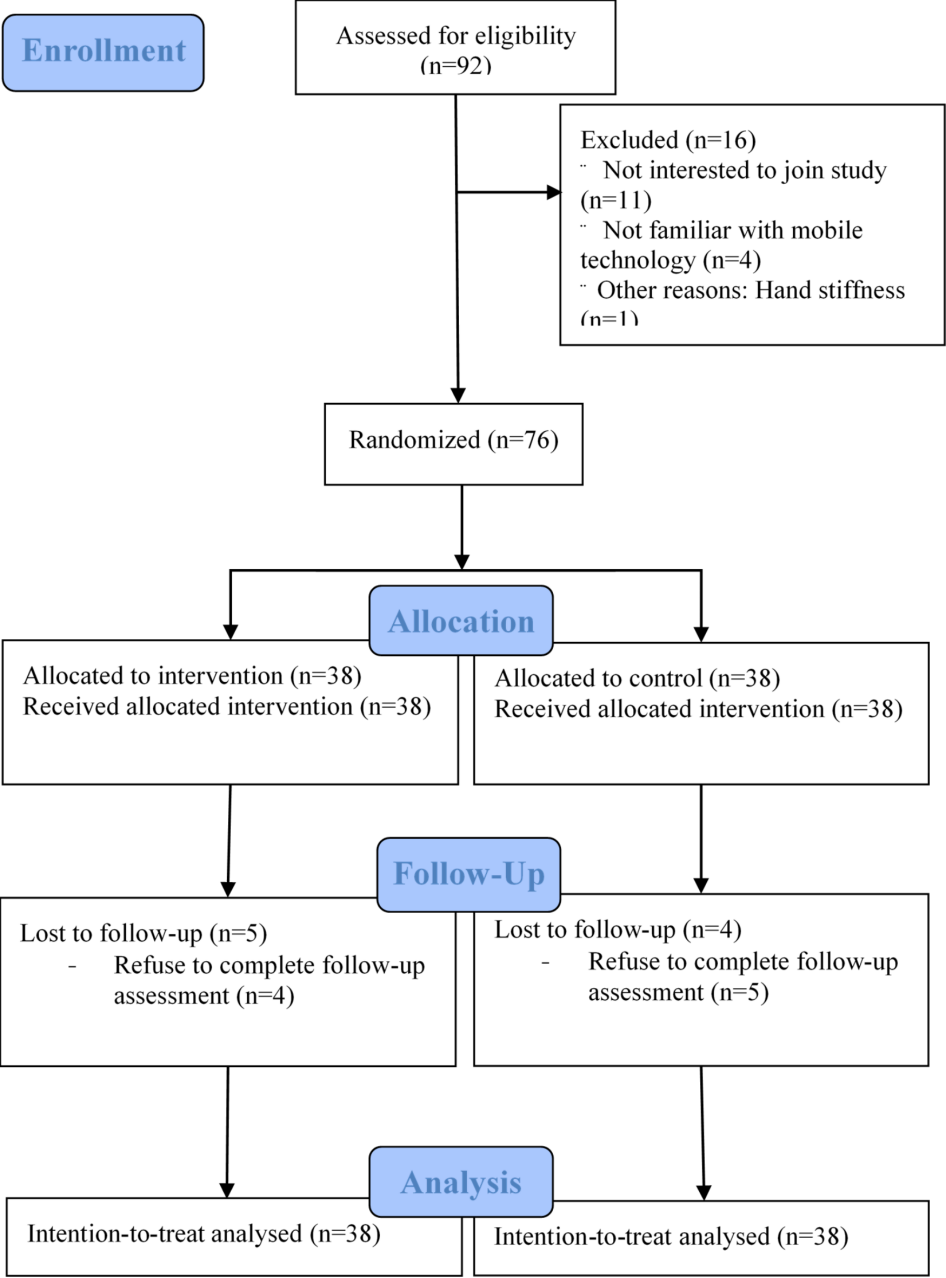
Flow chart for study recruitment and randomization.

The study was conducted from 12th August 2021 to 8th December 2022. The recruitment rate was 82.6% with 76 out of 92 eligible patients were recruited for the study (Fig. 1). The participants were randomly allocated to either the control or intervention group containing 38 participants each. Their mean age was 62.1 ± 8.6, with 63.2% being female and 36.8% being male (Table 1). Their mean duration of finger symptoms before injection was 4.4 ± 2.9 months. Most of the participants had underlying chronic illness (82.9%), including hypertension, hyperlipidaemia, diabetes, cardiovascular diseases, thyroid disease and cancer. Around 44.7% of the participants were employed, while the others were unemployed (5.3%), retired ( 21.1%) or homemakers (28.9%). Approximately 9.2% of the participants had received a previous steroid injection of the same finger. The participants’ mean NPRS was 1.3 ± 5.5, mean Quickdash score was 15.6 ± 13.7, and 65.8% of them had Quinelle grade 0 at recruitment. Baseline characteristics had no statistically significant difference except for the duration of finger symptoms in the intervention group (5.2 ± 2.9 months) was significantly longer than that of the control group (3.8 ± 2.6 months) (*P* = 0.002).

At 24 weeks of the study, 34 (89.5%) of the participants in the control group and 33 (86.8%) in the intervention group responded to the online survey, respectively. The follow-up rate of the study was 88.2%.

There was no significant difference in NPRS (*P* = 0.209) between the intervention and control groups at 24 week after controlling for age, sex and the duration of symptoms as covariate (Table 2). There was also no significant difference in the change of Quinelle grading (*P* = 0.697) between the 2 groups (Table 3). Among those who responded to the online survey, there was no significant difference in the self-rated change of finger condition (*P* = 0.695) (Table 4). The recurrence of trigger finger was reported in 22 (66.7%) of the intervention group and 24 (70.6%) in the control group, with no statistically significant difference (*P* = 0.997). In the intervention group, 7 (21.2%) participants required repeated trigger finger steroid injection, while 4 (11.8%) from the control group required repeated injection, though the difference was not statistically significant (*P* = 0.658). New occurrences of trigger finger were reported in 5 (15.2%) of the participants in the intervention group and 4 (11.8%) in the control group, the difference was not statistically significant ( *P* = 1.00). There was no report of any adverse effect from the finger exercises in the intervention group.

**Table 1:** Demographic background (mean ± standard deviation (SD) / count (col %)).

			Entire (*n* = 76)	Intervention (*n* = 38)	Control (*n* = 38)	Statistical value	*P* value
Age (years) ± SD			62.1 ± 8.6	61.0 ± 8.8	63.1 ± 8.3	1.059	0.293 ^a^
Female (%)			48 (63.2%)	28 (73.7%)	20 (52.6%)	3.619	0.057 ^c^
Finger involved (%)							
	Right	thumb	6 (7.9%)	2 (5.3%)	4 (10.5%)	3.766	0.866 ^d^
		2nd	2 (2.6%)	1 (2.6%)	1 (2.6%)		
		3rd	22 (28.9%)	13 (34.2%)	9 (23.7%)		
		4th	16 (21.1%)	8 (21.1%)	8 (21.1%)		
		5th	0 (0%)	0 (0%)	0 (0%)		
	Left	thumb	7 (9.2%)	4 (10.5%)	3 (7.9%)		
		2nd	2 (2.6%)	1 (2.6%)	1 (2.6%)		
		3rd	16 (21.1%)	8 (21.1%)	8 (21.1%)		
		4th	5 (6.6%)	1 (2.6%)	4 (10.5%)		
		5th	0 (0%)	0 (0%)	0 (0%)		
Symptoms duration (months) ± SD			4.4 ± 2.9	5.2 ± 2.9	3.6 ± 2.6	−3.086	0.002 ^b^*
Chronic illness (%)			63 (82.9%)	30 (78.9%)	33 (86.8%)	0.835	0.361 ^c^
Work status (%)							
	Employed		34 (44.7%)	18 (47.4%)	16 (42.1%)	3.446	0.348 ^d^
	Unemployed		4 (5.3%)	3 (7.9%)	1 (2.6%)		
	Retired		16 (21.1%)	5 (13.2%)	11 (28.9%)		
	Homemakers		22 (28.9%)	12 (31.6%)	10 (26.3%)		
Previous injection (%)			7(9.2%)	2 (5.2%)	5 (13.2%)	-	0.430 ^e^
Pre-injection							
NPRS ± SD			6.72 ± 2.2	6.50 ± 2.3	6.95 ± 2.0	−0.877	0.381 ^b^
Quinelle (%)							
	0		1 (1.3%)	0 (0%)	1 (2.6%)	1.565	0.843 ^d^
	1		3 (3.9%)	1 (2.6%)	2 (5.3%)		
	2		52 (68.4%)	26 (68.4%)	26 (68.4%)		
	3		20 (26.3%)	11 (28.9%)	9 (23.7%)		
Quickdash ± SD			37.0 ± 17.4	38.5 ± 19.7	35.6 ± 14.9	−0.715	0.477 ^a^
Post-injection							
NPRS ± SD			1.3 ± 5.5	1.9 ± 7.7	0.7 ± 0.9	−0.251	0.802 ^b^
Quinelle (%)							
	0		50 (65.8%)	24 (63.2%)	26 (68.4%)	0.891	0.708 ^d^
	1		23 (30.3%)	13 (34.2%)	10 (26.3%)		
	2		3 (3.9%)	1 (2.6%)	2 (5.3%)		
Quickdash ± SD			15.6 ± 13.7	16.8 ± 14.4	14.4 ± 13.0	−0.700	0.484 ^b^
^a^, Independent t-tests were used; ^b^, Mann-Whitney tests were used; ^c^, Pearson Chi-square tests were used; ^d^, Fisher-Freeman-Halton Exact Tests were used; ^e^, Fisher’s Exact Test was used; *, *p* < 0.05							

**Table 2:** Change of NPRS between groups by times (n=76)).

	Univariate model ^a^		Multivariate model ^b^	
	β (95% CI)	P value	β (95% CI)	P value
Age	0.036 (−0.048, 0.120)	0.397	0.032 (−0.053, 0.116)	0.462
Sex	−0.277 (−1.767, 1.214)	0.715	−0.673 (−2.215, 0.870)	0.392
Duration of symptoms	−0.208 (−0.447, 0.031)	0.089	−0.217 (−0.474, 0. 039)	0.097
NPRS	0.539 (−0.249, 1.327)	0.180	0.508 (−0.285, 1.301)	0.209
Intervention group	−0.503 (−1.925, 0.919)	0.487	−0.237 (−1.731, 1.257)	0.755
Abbreviate: β = beta; CI = confidence interval; NPRS = Numeric Pain Rating Scale; ^a^. adjusting for NPRS at baseline; ^b^. adjusting for NPRS at baseline, age, sex, and the duration of symptoms.				

**Table 3:** Change in Quinell grading across the period of the study (n=76).

		Intervention (*n* = 33) ^d^	Control (*n* = 34) ^d^	OR (95% CI) ^a c^	*P* value	OR (95% CI) ^a b c^	*P* value
Quinell grading							
	No change/Worsen	29 (87.9%)	30 (88.2%)	Ref.		Ref.	
	Improved	4 (12.1%)	4 (11.8%)	1.083 (0.266, 4.405)	0.911	0.730 (0.150, 3.556)	0.697
Abbreviate: CI = confidence interval; OR = odd ratio; Ref.=reference ^a^. Participants showing “No change/Worsen” on Quinell grading were set as the reference group. ^b^. adjusting for age, sex and duration of symptoms; ^c^. the imputed datasets were used for the analysis; ^d^. the dataset that included missing values was counted for both groups.							

For the intervention group, the overall exercise log response rate was 85.6% and the response rate was highest at the 4th week (93.9%) ( Table 5). The overall finger exercise compliance rate was 68.6% over the 24 weeks study period and the compliance rate was highest at the 4th week (78.6%).

**Table 4:** Comparison on number of repeated injection, Occurrence of new trigger finger and self-rated change of finger condition (n=76).

		Intervention (*n* = 33) ^d^	Control (*n* = 34) ^d^	*P* value
Self-rated change of finger condition ^b^				
	Improved	15 (45.5%)	12 (35.3%)	0.695
	No change	1 (3.0%)	1 (2.9%)	
	Worsen	17 (51.5%)	21 (61.8%)	
Recurrence of same finger^c^				
	Yes	22 (66.7%)	24 (70.6%)	0.997
	No	11 (33.3%)	10 (29.4%)	
Repeated finger injection^c^				
	Yes	7 (21.2%)	4 (11.8%)	0.658
	No	26 (78.8%)	30 (88.2%)	
Occurence of new trigger finger ^a^				
	Yes	5 (15.2%)	4 (11.8%)	1.000
	No	28 (84.8%)	30 (88.2%)	
^a^. Fisher Exact Tests was used; ^b^. Fisher-freeman-Halton Exact Test was used; ^c^. Pearson chi-square test was used; ^d^. the dataset that included missing values was counted for both groups.

**Table 5:** Exercise log response rate and compliance rate from intervention group (mean ± standard deviation (SD) / count (col %)).

Exercise log no.	1	2	3	4	5	6	7	8	Total
Week	1	2	3	4	8	12	16	20	
No. of response to online exercise log N=33	25	30	30	31	28	28	28	26	28.6±2.2
Response rate (%)	75.8	90.9	90.9	93.9	84.8	84.8	84.8	78.8	85.6
Mean no. of exercise entries N=14	8.6 ±5.7	10.4±4.4	10.5±4.5	11.0±4.1	9.8 ±5.4	9.3 ±5.6	8.9 ±5.5	8.5 ±5.5	9.6 ±5.1
Compliance rate (%)	61.4	74.3	75.0	78.6	70.0	66.4	63.6	60.7	68.6

## Discussion

Our study showed no significant difference in NPRS and Quinnell grading between the intervention and control group. There was also no statistically significant difference in the rate of trigger finger recurrence, rate of repeated injection and rate of new trigger finger occurrence of both groups at 24 weeks.

Our study had a satisfactory follow-up rate of 88.2%. For the intervention group, the exercise log response rate was good at 85.6%. We required participants in the intervention group to do the finger gliding exercises 2 times per day for 24 weeks and the finger gliding exercises response rate was 68.6%. We expect a decrease in exercises compliance over time, but the response rate was maintained at 60.4% even at week 20. We think that it is a satisfactory response and most participants had done the exercises at least once per day. For a therapeutic exercise to be effective, the compliance to the exercises is very important^25^. Our exercise monitoring method, by means of online exercise logs, appears to be user-friendly. As Hong Kong has high household internet access rate (96.1%) and smartphone penetration rate ( 97.1%), making this online method a feasible and convenient way to monitor exercise compliance among our population^26^. Also, our exercise is easy to perform and participants are able and willing to do it consistently for at least 24 weeks.

Our study did not demonstrate beneficial effect of finger gliding exercise compared to usual care, with an effect size of only 0.17 for pain score reduction. Actually, this finding aligns with most studies on finger tendon gliding exercise in patients with CTS, which have shown effectiveness only when performed in conjunction with other conservative treatments^20,27,28^. However, our intervention group had longer symptom duration before injection, which is a risk factor for poorer recovery. Studies have shown that symptoms persisting over 6 months are associated with lower success rates after steroid injection^29–31^. This may partly explain the lack of significant results.

Nevertheless, one potential reason for our study not showing beneficial effect of finger gliding exercises might be the complex and not fully understood pathophysiology of trigger fingers^32^. Histopathological studies of trigger fingers have revealed pathological changes such as tendinosis with synovial reaction and inflammation, cartilaginous metaplasia of pulleys with increased fibrous thickening, edematous extracellular matrix, and increased hyaluronic acid synthesis^33,34^. Finger tendon gliding exercises focus on improving mechanical gliding of tendons, but may not be sufficient to halt the progression of pathology.

**Fig. 2:**
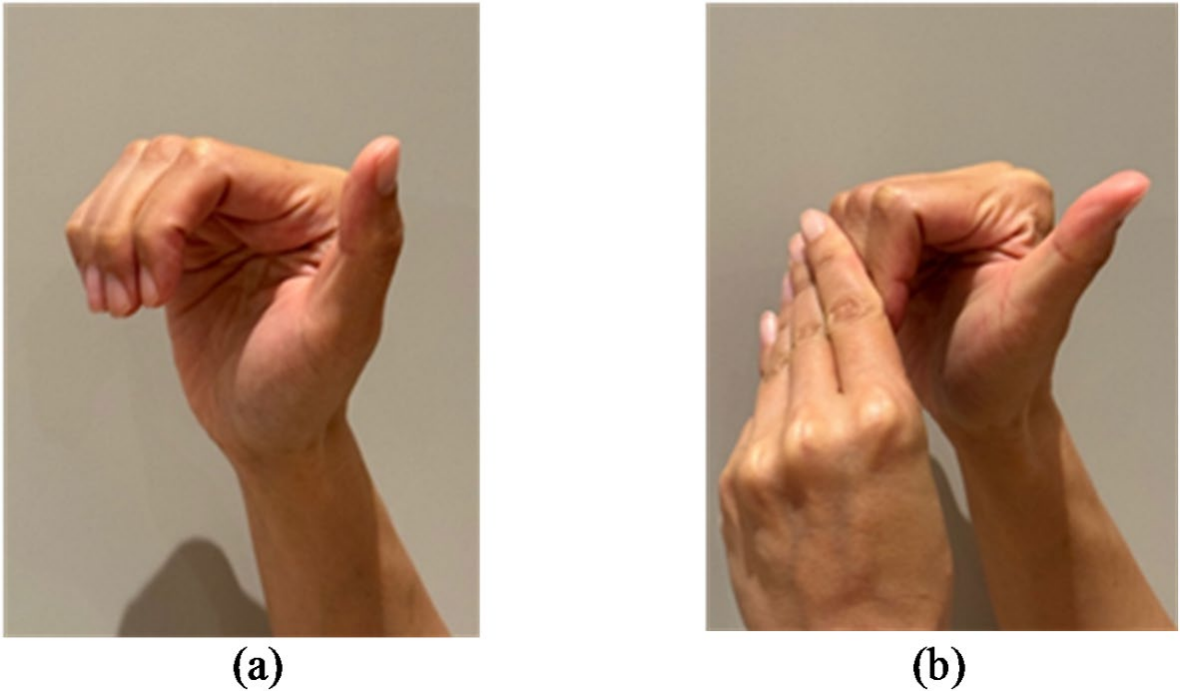
Completing the straight fist position with the help of another hand **a** Participant with difficulty in performing the straight fist exercise due to finger tightness **b** Participant using the other hand to help completing the straight fit position.

Furthermore, the accuracy of performing finger gliding exercises may also influence the study outcomes. In our study, some participants found it technically challenging to perform the straight fist position, which requires straightening the distal interphalangeal joint while keeping the metacarpophalangeal joint at a 90-degree angle. To address this, we often instructed participants to use their other hand to assist in completing the position (Fig. 2). Following our guidance, all participants were able to perform the exercise in the correct position. Since our study did not supervise the finger exercises or reassess the finger condition of participants in person at the study’s conclusion, we cannot confirm whether participants achieved improved finger motility through the exercises. If the exercises are not performed accurately, their intended effect of maximizing tendon gliding may not be achieved. This problem may also be present in other studies related to finger tendon gliding exercises, although we could not identify any studies specially addressing this problem or assessing the accuracy of the exercise performance.

Our study results should be interpreted with the consideration of the response and recurrence rates associated with steroid injections. Steroid injection is widely recognized as an effective treatment options for trigger finger^24,35^. Long-term successful rate of corticosteroid injection varies between 40 and 90%^36^, while recurrences reported between an average of 3 and 37 months after initial treatment^37^. Notably, a substantial number of recurrences develop after 6 months following injection^13,38,39^. Sheikl et al. found injection failure rate increased dramatically from 22% at 6 months to 54% at 1 year^38^, while Leow et al. reported 60% symptom resolution at 12 weeks, but a recurrence rate of 56% between 12 and 24 weeks^39^. On the other hand, Seignerman D. et al. evaluated the short-term patient response to corticosteroid injections and observed that most patients experienced symptom relief by the third week after injection^40^.

In our study, to ensure fair comparison, we recruited all patients during follow-up visits 4–6 weeks post-injection, a point at which the full effect of the steroid injection was expected to have taken place. Also, all the participants received injection from the same physician. However, as participants only began practicing the finger gliding exercisese 4–6 weeks post-injection, we cannot determine whether the exercises would have been more effective if initiated earlier after the steroid injection.

Additionally, we assessed recurrence for trigger finger symptoms only at 24 weeks. As a result, we could not compare the effects of finger gliding exercises among participants with different recurrence rates after steroid injection. Given the negative findings of our study result at 24 weeks, we believe that a duration of 12 weeks might be insufficient to identify recurrence, as symptoms often become apparent between 6 and 12 months. Repeated assessment of recurrence at 9 months and 1 year could provide a better evaluation of the effect of finger gliding exercises. However, maintaining participant compliance with the exercises over such a prolonged study period could pose a challenge.

One of the limitation of our study is the absence of functional outcome measures, such as grip strength, dexterity, or hand-specific disability scores. Notably, most intervention studies on trigger finger have utilized symptom resolution as the outcome measure rather than assessing hand function^41,42^. Langer D et al. suggested that this trend may be attributed to the lack of validated hand function assessment specifically designed for trigger finger^41^. In our study, the QuickDASH score^43^, a self-reported tool designed to measure physical function and symptoms in individuals with upper limb musculoskeletal disorders, was used solely to compare the baseline characteristics of the study groups and not as an outcome measure. This decision was due to the potential variability in QuickDASH results if participants developed other upper limb conditions, making it less specific to the functional impairments of a single affected digit. Additionally, our study relied on remote data collection via online surveys, which was particularly advantageous given that it was conducted during the COVID-19 period. However, this approach posed logistical challenges for conducting a comprehensive evaluation of hand function. Also, we were concern that including more detailed assessments into the online survey might reduce the participant response rates and compromise data quality. Nevertheless, we acknowledge the importance of functional assessment. According to the study by Tung et al. on kinemetic and functional differences in different graded trigger fingers, patients diagnosed within the same grade showed a large variation of movement patterns or symptoms among each other^42^. These findings highlight the limitations of relying solely on clinical grading systems, such as Quinell grading, to capture the full scope of functional impairments. Future studies should consider incorporating functional assessment to provide a more comprehensive understanding of the impact of finger gliding exercises on patients’ daily functioning and quality of life.

Our study has other limitations. The finger exercises were not performed under supervised conditions. The exercise log relied solely on self-reported retrospective record of finger exercises, making it susceptible to recall bias. Additionally, for the survey at 24 weeks, Quinnell grading of fingers was based on patient’s self-assessment only, which could impact the accuracy of the assessment. However, we consider participants’ self-reported improvement or recurrent of symptoms as a more important assessment criterion in our study. Furthermore, occupations and hobbies involving repetitive hand movements and prolonged grasping may increase the risk of trigger finger^44^. Our study did not explore the exact work or daily hand movement patterns of the participants. Lastly, our study excluded patients without smartphones, internet access, or proficiency in Chinese, potentially underrepresenting individuals from remote areas or with lower socio-economic or educational levels. This limits the generalizability of our findings to these populations.

To the best of our knowledge, this study is the first to explore the effectiveness of finger gliding exercises in patients with trigger finger. Finger gliding exercises are commonly included in the management programme for trigger fingers, along with other nonconservative therapeutic modalities such as heat, massage and splinting^22–24^. Many patients with trigger finger are eager to learn techniques to prevent symptom recurrence after steroid injections. It is not uncommon for them to encounter finger gliding exercises when seeking information in online resources for trigger finger, and may practice these exercises independently^45^. The novelty of our study lies in its systemic investigation of this simple, commonly recommended yet understudied intervention for trigger finger. The gap in research highlights the need for evidence-based management to ensure that the recommendations provided to patients are both effective and safe. While our results did not demonstrate clinical effectiveness of finger gliding exercises, factors such as symptom duration, exercise timing, outcome measurement, and performance accuracy may have influenced the results. Further research incorporating functional assessments and exploring the combined use of finger gliding exercises with other conservative modalities is needed to better evaluate the potential benefits of finger gliding exercises for trigger finger.

## Conclusion

This study showed that there was no statistical significant improvement in outcomes for patients who performed regular finger tendon exercises following steroid injections for their trigger finger compared to those receiving usual care. Further study trials are warranted to further investigate the clinical usefulness of finger gliding exercises.

## Method

### Study design

It was a prospective, two-arm, 1:1 randomized controlled study conducted over a 68 weeks period, with outcomes assessed through a 24-week online survey. It was approved by the Joint Chinese University of Hong Kong and New Territories East Cluster (CUHK-NTEC) Clinical Research Ethnics Committee (CREC) (ref: 2021.251) and was registered in the International Standard Randomised Controlled Trial Number (ISRCTN) registry (ISRCTN57109334, registration date: 03/04/2024). The study protocols were approved by the CUHK-NTEC CREC and the study was carried out in accordance with relevant guidelines and regulations. Written informed consents were obtained from all the study participants.

### Setting and participant

The study was conducted at the family medicine musculoskeletal (MSK) clinic at Prince of Wales Hospital, Hospital Authority, Hong Kong. All patients who received corticosteroid injection for trigger finger at the MSK clinic were scheduled for a follow-up appointment 4–6 weeks post-injection. Patients attending the follow-up visits were invited to participate in the study. Inclusion criteria included: patients aged ≥ 18; had a single finger triggering at presentation; had received a corticosteroid injection within 4–6 weeks, and having a post-injection Quinelle grading of 2 or below^46^. Exclusion criteria included: patients who did not have a smartphone, no internet access, and could not read or understand Chinese.

### Intervention group with video-based exercise prescription

The finger gliding exercises prescription for the intervention group encompassed the exercises described by Wehbe and Hunter^19^. It included exercises at 3 basic fist positions: (1) straight-fist position: the distal interphalangeal joint (DIPJ) extends while the metacarpophalangeal joint (MCPJ) and proximal interphalangeal joint (PIPJ) are flexed; (2) hook: MCPJ extends while DIPJ and PIPJ are flexed; (3) full fist: all the finger joints are fully flexed The straight-fist position provided maximum FDS excursion while the full fist position provided maximum FDP excursion. The hook position allowed maximum differential gliding by providing more FDP than FDS excursion. As the thumb only involved one flexor tendon FPL, maximum gliding could be achieved by flexing the interphalangeal joint and MCPJ. Each fist position exercise was to be done with 10 repetitions each time. Participants were suggested to do the finger gliding exercises 2 times per day (Fig. 2). A YouTube link ( https://youtu.be/g4exjvULYIU?si=s5AX_WFcKAXDvGmr ) demonstrating the exercises was prescribed to the participants.

### Control group with usual finger care

Participants from the control group were given an education leaflet on general finger care, which included advice on finger protection, gentle hand stretching and massage; however, finger gliding exercises were not included.

### Outcome measures

The data of outcome measures was collected at baseline and at 24 weeks post intervention. Pain being a primary symptom of trigger finger, its reduction is often the primary goal of treatment^47^. Therefore, the primary outcome was the overall finger pain severity at 24 weeks, measured by the Numerical Pain Rating Scale (NPRS 0–10). The secondary outcomes included other clinical measures such as the Quinelle grading, self-rated change of finger condition, recurrence of triggering of the same finger, number of repeated injections, occurrence of new trigger finger, and compliance with finger gliding, assessed by measuring the exercise log response and compliance rate. The self-rated change of finger condition was measured by asking patients to indicate whether they had improved, no change, or worsen of symptoms. Quinelle grading classified trigger finger based on severity of symptoms and was rated from grade 0 (normal) to grade 4 (locked finger). Participants who had self-rated worsen finger condition or who had repeated injection were regarded to have recurrence. The compliance to finger gliding exercises was monitored with online exercise log. The online exercise logs were sent to participants weekly for the first 4 weeks of the study and then 4 weekly till the end of the study. All participants in the intervention group were requested to submit 8 exercise logs in total. The exercise log requested patients to have recalled exercise entries for the past 1 week. As participants were requested to do the finger gliding exercise twice per day, there were 14 exercise entries per exercise log. Exercise log response rate was the percentage of online exercise logs submitted. The exercise compliance rate was the percentage of exercise entries recorded.

### Sample size

There had been no previous study on the evaluation of the effectiveness of finger gliding exercises for trigger fingers. A study that compared the effectiveness of finger gliding exercises with nerve gliding exercise for patients with carpal tunnel syndrome (CTS) found that finger gliding exercises (together with splint and paraffin therapy) had an effect size of 0.78 for symptom severity and 0.96 for pain scale score^27^. Taking this finding as reference, we assumed an effect size of 0.7 for the study. With an effect size of 0.7, the a power analysis of a two-sample t test indicated that the minimum sample size to yield a statistical power of at least 80% and a 2-sided type I error rate of 5% was 34 participants. The sample size was calculated using G*Power software with the following formula^48^:

$$\delta = d \times\:{}\sqrt\frac{\eta1\eta2}{\eta1 + \eta2}$$

Where:

- *d* = 0.7: Effect size.
- η1 = η2 = 34: Sample size per group.
- α = 0.05: Two-sided type I error rate.
- Power = 80%.

Presuming a 10% attrition rate, an addition of 4 participants was recruited in each group, making a total of 38 participants in each group.

### Randomization, allocation, concealment and blinding

An off-site research assistant performed 1:1 randomization to allocate the participants into 2 groups. The allocation sequence was concealed from the researcher and patients using sequentially numbered, opaque, sealed envelopes. The corresponding envelopes were opened at the time of intervention assignment by the principal investigator after all the enrolled patients had undergone all baseline assessments. In this open-label study, blinding of patients was not possible. However, research assistants involved in data collection and analysis, respectively, were blinded to the allocation status.

### Data collection

Data collection at baseline and 24 weeks.

Baseline data collection included patient demographic, medical background, NPRS, Quinelle grading and Quickdash score. All the participants were expected to answer the online survey for the progress of finger condition at 24 weeks from recruitment and the outcome measurement included self-rated change of finger condition, NPRS, Quinelle grading, record of repeated finger injection, and occurrence of new trigger finger site. The online survey was sent through SMS (short message service).

### Exercise compliance by online exercise log

Intervention group participants’ compliance to gliding exercises was collected from online exercise logs which were sent through SMS. The exercise log requested participants to have recalled exercise entries for the past 1 week on an online calendar. There should have been 14 exercise log entries per week for a compliance rate of 100%. The exercise log was sent weekly for the first 4 weeks of the study and then 4 weekly till the end of the study. Besides for exercise record, the exercise log also intended to be a reminder for exercises.

### Statistical analysis

Demographic information of participants was presented as the mean ± standard deviation (SD) for continuous variable and as the percentage for categorical variables. The normality of continuous variables was examined using Shapiro-Wilk testing. The independent t-tests, Mann-Whitney tests or Chi-square tests were used to compare the difference between groups at baseline. Variables found to be significantly different at baseline were adjusted for in the subsequent analyses. Intention-to-treat (ITT) analysis was conducted for the primary and secondary outcomes. Missing data were imputed using chained equations with predictive mean matching for continuous variables and logistic regression models for categorical variables separately. Analysis of covariate analysis (ANCOVA) was used to compare the effects of intervention versus control on NPRS, adjusting for age, sex, symptoms duration, and NPRS baseline scores. The change in Quinelle grading between groups was compared using logistic regression, adjusting for age, sex and symptoms duration. Variables such as the occurrence of new trigger finger site, the number of repeated injections, and the self-rated change of finger condition were analyzed using the Chi-square test. The same analysis for the primary outcome was conducted using the dataset that contained missing data for sensitivity analysis ( Supplementary Table S1 ). A value of *p* < 0.05 was considered statistically significant. All analyses were conducted using SPSS version 27 (Statistical Package for the Social Science) and R version 4.4.1.

## Supplementary Information

Below is the link to the electronic supplementary material.

Supplementary Material 1

## Data Availability

The datasets used and/or analyzed during the current study available from the corresponding author on reasonable request.
