# Supplementary material for: Clinical effectiveness of Finger gliding Exercise for patients with trigger fingers receiving steroid injection: a Randomized Clinical Trial
Source: Sci Rep. 2025 Feb 11;15:5141. doi: 10.1038/s41598-025-89436-9 (PMC11814069; doi:10.1038/s41598-025-89436-9)
Supplement: Supplementary file 1 — Supplementary Material 1 [file 41598_2025_89436_MOESM1_ESM.docx]

| Supplementary Table S1. Change of NPRS between groups by times (n=67) ^c^ | | | | |
| --- | --- | --- | --- | --- |
|  | Univariate model ^a^ | | Multivariate model ^b^ | |
|  | β (95% CI) | P value | β (95% CI) | P value |
| Age | 0.045 (-0.047, 0.137) | 0.331 | 0.038 (-0.055, 0.130) | 0.421 |
| Sex | -0.008 (-1.569, 1.554) | 0.992 | -0.391 (-1.989, 1.207) | 0.627 |
| Duration of symptoms | -0.255 (-0.500, -0.010) | 0.042* | -0.259 (-0.523, 0.004) | 0.053 |
| NPRS | -0.018 (-0.147, 0.111) | 0.781 | -0.012 (-0.140, 0.117) | 0.858 |
| Intervention group | -0.504 (-2.003, 0.994) | 0.504 | -0.098 (-1.652, 1.456) | 0.900 |
| Abbreviate: β=beta; CI=confidence interval; NPRS= Numeric Pain Rating Scale;  ^a^. adjusting for NPRS at baseline;  ^b^. adjusting for NPRS at baseline, age, sex, and the duration of symptoms;  ^c^. the dataset that contains missing data was used. | | | | |
